# Impact of different surgical and postoperative adjuvant treatment modalities on survival of sinonasal malignant melanoma

**DOI:** 10.1186/1471-2407-14-608

**Published:** 2014-08-23

**Authors:** Xin-Jun Meng, Hua-Fei Ao, Wei-Ting Huang, Fu Chen, Xi-Cai Sun, Jing-Jing Wang, Zhuo-Fu Liu, Wade W Han, Allison N Fry, De-Hui Wang

**Affiliations:** Department of Otolaryngology-Head and Neck Surgery, Eye Ear Nose and Throat Hospital, Fudan University, Shanghai, 200031 China; Department of Otolaryngology, Shanghai 3rd People’s Hospital, School of medicine, Shanghai Jiao Tong University, Shanghai, 201900 China; Department of Radiation Oncology, Eye Ear Nose and Throat Hospital, Fudan University, Shanghai, 200031 China; Florida Ear Nose Throat and Facial Plastic Surgery, Orlando, Florida 32819 USA; Department of Otolaryngology, Ruijin Hospital, School of medicine, Shanghai Jiao Tong University, Shanghai, 200025 China

**Keywords:** Sinonasal, Melanoma, Prognosis, Endoscopic sinonasal surgery, Radiotherapy, Chemotherapy

## Abstract

**Background:**

The role of postoperative adjuvant treatment for sinonasal malignant melanoma remains unclear. This study evaluates the impact of three different surgical and postoperative adjuvant treatment modalities: surgery alone(open and endoscopic approaches), surgery plus radiotherapy and surgery, radiotherapy plus chemotherapy on survival of patients with primary sinonasal malignant melanoma (SMM).

**Methods:**

The data of 69 patients who underwent primary surgical treatments at Eye & ENT hospital of Fudan University between January 1st, 2000 and December 31st, 2010 were retrospectively reviewed. Survival comparison of different surgical and postoperative adjuvant treatment modalities (surgery alone, surgery plus radiotherapy and surgery, radiotherapy plus chemotherapy), as well as survival comparison between open and endoscopic surgical approaches were performed. Curves depicting survival were performed using Kaplan-Meier method. Statistical analysis was performed using log-rank test software SPSS19 and p < .05 is considered as statistically significant.

**Results:**

The median overall survival time was found to be 18 months for surgery alone (27 cases), 32 months for surgery plus radiotherapy (24 cases), 42 months for surgery, radiotherapy plus chemotherapy (18 cases). The 3 and 5 year survival rates for groups mentioned above were 14.8% and 5.6%, 45.1% and 31.6%, 55% and 32.1%, respectively. Statistical significances were found not only between surgery alone and surgery plus radiotherapy treatment group (P = 0.012), but also surgery alone and surgery, radiotherapy plus chemotherapy group (P = 0.002). There was no statistically significant survival difference found between the two different surgical approaches (41 cases for open approach and 28 cases for endoscopic approach).

**Conclusions:**

Sinonasal malignant melanoma is a disease with a poor prognosis. Patients who underwent surgery plus radiotherapy or surgery, radiotherapy plus chemotherapy had better survival outcomes than those underwent surgery alone. Endoscopic approach provided similar survival outcome as an open approach.

## Background

Malignant melanoma is a neoplasm consisted of aberrant melanocytes which originate from neural crest cells. Mucosal malignant melanoma arising from the nasal cavity and the paranasal sinuses only accounts for 0.3-2% of all malignant melanomas and approximately 4% of head and neck melanomas [1], with a poor prognosis of 5-year overall survival rate ranging between 20-43% [2–6].

Common consensus is that complete tumor resection is the main stay of therapy for sinonasal malignant melanoma(SMM) [1]. However, sometimes complete tumor excision with clear surgical margins may not be achievable due to limited surgical visualization, anatomical complexity of sinonasal region and involvement of adjacent vital structures. Therefore, adjuvant radiotherapy and chemotherapy are often planned and performed postoperatively. Although there were reports that adjuvant radiotherapy could benefit local control [7, 8], and chemotherapy could benefit overall survival of mucosal malignant melanoma [9], the role of radiotherapy and chemotherapy remains unclear.

Before the advent of nasal endoscopy, sinonasal malignancies, especially the extensive ones, were most frequently resected through open approaches such as lateral rhinotomy, midfacial degloving or tranpalatal resection. Recently more patients have been treated with an endoscopic approach, owning to the advantages of its minimally invasiveness, direct tumor exposure, more desirable cosmetic appearance and shorter hospital stay. Since there is a low incidence of sinonasal malignant melanoma, little is known about the impact of open vs. endoscopic surgical approaches on the survival of patients with SMM.

SMM is rare and most related studies were case reports or retrospective analysis of data from series of patients over many decades. The oncologic results for endoscopic resection of SMM have rarely, if any, been reported. Many published data consisted of heterogeneous histopathologic findings.

In this report, we reviewed clinical data from 69 patients with a homogeneous histopathologic diagnosis of malignant melanoma who underwent primary surgical treatments at our department between January 1st, 2000 and December 31st, 2010. We compared the overall, cause-specific and disease-free survival rate through various surgical and postoperative adjuvant treatment modalities (surgery alone, surgery plus radiotherapy and surgery, radiotherapy plus chemotherapy), as well as between open and endoscopic surgical approaches.

## Methods

A retrospective review was performed to analyze data from patients who underwent primary surgical treatments at Eye Ear Nose and Throat hospital of Fudan University between January 1st, 2000 and December 31st, 2010. This study was approved by the institutional review board of Fudan University. All together, 69 patients were included in this study who met the clinical and histopathological criteria of sinonasal malignant melanoma. In addition to those tumors originating from other sites outsides sinonasal region and/or those had metastasized from elsewhere in the body, patients whose primary surgeries that were not performed at this hospital were also excluded.

Clinical information retrieved included demographic data, chief symptoms, duration of symptoms before diagnosis, staging, surgical treatment, adjuvant therapies, overall survival time, locoregional control rate, disease-specific as well as disease-free survival status. The predominant sites of tumor were also recorded. For tumors involving structures of lateral nasal wall such as turbinates, meatus or uncinate process were recorded as lateral nasal wall. When tumors were too extensive in the nasal cavity to ascertain their origin, then they were recorded as nasal cavity.

According to the seventh editions of the AJCC cancer staging manual and handbook for mucosal melanoma of the Head and Neck [10], patients were staged by clinical manifestations, CT/MRI and nasal endoscopy findings as well as histopathology on biopsy.

Through various surgical treatment modalities, patients were classified into three groups: surgery alone(sa), surgery plus radiotherapy (sr), surgery, radiotherapy plus chemotherapy(src). According to surgical approaches, they were also grouped into open and endoscopic groups. Curves depicting survival were performed using Kaplan-Meier method. Statistical analysis was performed using log-rank test software SPSS19 and p < .05 is considered as statistically significant.

More recent surgical resection of SMM were mostly performed endoscopically by senior author (De-Hui Wang), using standard Kennedy Functional Endoscopic Sinus Surgery (FESS) approach. Once the tumor invades the nasal septum, then septectomy would be performed endoscopically. For tumors involving the orbit while orbital periosteum still intact, ethmoidectomy including lamina papyracea would be performed. For large tumors invading the skull base, medial maxillectomy, ethmoidectomy and sphenoidectomy were performed successively. Then the posterior wall of the maxillary sinus was removed in order to open pterygopalatine fossa. After identifying the opening of the vidian canal at the base of the pterygoid plates, the bones of vidian canal can be removed cautiously along its inferior and medial aspect. Vidian nerve can be used as an important landmark to identify the anterior genu of the internal carotid artery. Once the internal carotid artery is identified, the tumor with the adjacent mucosa and bone can be removed carefully. Intraoperative navigation was used frequently to help identify vital structures and determine the border of tumor together with the resection range. Doppler ultrasound was also used to ascertain the location of internal carotid artery during the surgical process.

As for patients who accepted radiotherapy, a 3 dimensional conformal radiation therapy (3D CRT) was performed. The treatment area were allocated depending on the extent of involvement of the tumors. Radiation dosage to the primary tumor site ranged from 48 to 72 Gy, using 1.9-2.0 Gy/fraction. Mean dose was 63.40 Gy. If the nasopharynx or choanal was involved, upper neck was prophylactically irradiated with a dose of 50-55 Gy. A combination utilization of DTIC, VCR and DDP was the common chemotherapy regimen. Dexamethasone and Ondansetron Hydrochloride were used to alleviate side effects such as vomiting.

## Results

### Patient demographics and clinical findings

In general, there were 69 patients included (37 males and 32 females) in the study, and the mean age at diagnosis was 65.9 years (age ranging from 28–89 years). The two most common chief complaints at presentation were epistaxis in 43 cases (62%) and nasal obstruction in 23 cases (33%). Other symptom such as epiphora, noticeable mass and nasal dryness, each was found in 1 case. The mean duration of symptoms before diagnosis was 5 months (ranging from 0.5-60 months).

**Figure 1:**
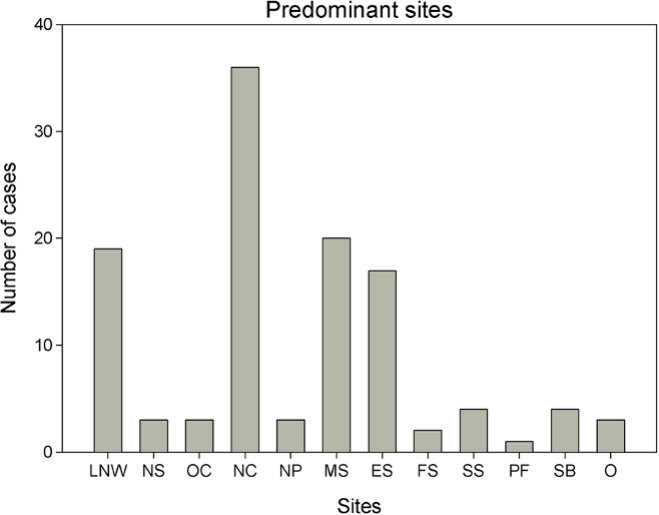
**Predominant sites of tumor invasion.**

The most common sites of tumor invasion is in the nasal cavity while the maxillary sinus is most common paranasal sinus invaded. Detailed information about predominant sites of tumor invasion is graphed in Figure 1.

According to AJCC cancer staging system, T classification was as follows: T3 37 cases (54%), T4a 27 cases (39%), T4b 5 cases (7%). Only 1 case suffered regional lymph node invasion at diagnosis. There were no cases associated with distant metastasis at diagnosis. Thus, 37 cases were classified as stage III, 27 as IVa, 5 as IVb. Clinical characteristics of 69 cases of sinonasal malignant melanoma are presented in Table 1.

**Table 1:** **Clinical Characteristics of 69 cases of Sinonasal malignant malenoma**

Case no.	Predominant sites	Chief complaint(duration,m)	Stage	Therapeutic modalities	Status/follow-up(m)
1	LNW	Epistaxis(3)	III	Lateral rhinotomy	Dead(9)
2	NP	Epistaxis(2)	IVa	approach from palatum	Dead(44)
3	OC	Epistaxis(1)	III	Lateral rhinotomy + R	Dead(29)
4	MS	Epistaxis(2)	IVa	Caldwell-Luc	Dead(1)
5	LNW	Epistaxis(2)	IVa	Lateral rhinotomy + R	Alive(144)
6	LNW	Epistaxis(1)	III	Lateral rhinotomy + R	Dead(18)
7	NC,MS	Epistaxis(0.5)	IVa	Lateral rhinotomy + R	Dead(10)
8	NC,MS,ES	Nasal obstruction(4)	III	Lateral rhinotomy	Alive(128)
9	LNW	Epistaxis(4)	III	Lateral rhinotomy + R	Dead(25)
10	LNW	Epistaxis(4)	III	Lateral rhinotomy	Dead(17)
11	MS,SB	Epistaxis(12)	IVb	Lateral rhinotomy + R	Alive(121)
12	NC,MS,ES,FS,SS,NP,PF	Nasal obstruction(6)	IVb	Lateral rhinotomy	Dead(21)
13	NS	Epistaxis(3)	III	ESS + R	Dead(49)
14	LNW	Nasal obstruction(12)	III	Lateral rhinotomy	Dead(15)
15	LNW	Epistaxis(1)	IVa	Lateral rhinotomy + R	Dead(12)
16	NC,MS	Epistaxis(6)	IVa	Lateral rhinotomy	Dead(6)
17	NC,ES,SS,O,SB	Nasal obstruction(4)	IVb	Lateral rhinotomy + R + C	Dead(50)
18	NC,ES	Epistaxis(2)	III	Lateral rhinotomy + R	Dead(10)
19	NC,ES	Nasal obstruction(15)	III	Lateral rhinotomy + R	Dead(43)
20	NC,MS,ES	Nasal obstruction(1)	IVa	Lateral rhinotomy + R	Dead(5)
21	LNW	Nasal obstruction(5)	III	Lateral rhinotomy + R	Alive(102)
22	LNW	Nasal obstruction(3)	III	Lateral rhinotomy + R	Dead(79)
23	NC	Epistaxis(4)	III	Lateral rhinotomy	Dead(24)
24	NC	Epistaxis(5)	III	Lateral rhinotomy	Dead(28)
25	NC,MS,SB	Epistaxis(2)	IVb	Lateral rhinotomy + R + C	Dead(59)
26	NC	Nasal obstruction(6)	III	Lateral rhinotomy	Dead(18)
27	LNW	Epistaxis(0.5)	III	Lateral rhinotomy + R	Alive(84)
28	MS,ES	Nasal obstruction(6)	IVa	Lateral rhinotomy + R	Dead(39)
29	NC	Epistaxis(3)	III	Lateral rhinotomy + R + C	Alive(81)
30	NC,ES,MS,O	Epistaxis(6)	IVa	Lateral rhinotomy + R	Dead(9)
31	NC,ES	Nasal obstruction(6)	IVa	Lateral rhinotomy + R	Dead(35)
32	LNW	Epistaxis(2)	III	Lateral rhinotomy	Dead(24)
33	LNW	Nasal obstruction(3)	IVa	Lateral rhinotomy + R + C	Dead(42)
34	NC,ES,MS,O	Mass(3)	IVa	Lateral rhinotomy + R	Dead(3)
35	NC	Epistaxis(1)	III	ESS + R + C	Alive(72)
36	LNW,ES	Epistaxis(3)	III	Lateral rhinotomy + R	Dead(5)
37	NC	Nasal obstruction(4)	III	Lateral rhinotomy + R	Alive(65)
38	NC	Epistaxis(6)	III	ESS + R + C	Alive(65)
39	LNW,MS	Epistaxis(3)	IVa	ESS	Dead(39)
40	NC,ES,MS	Epistaxis(1.5)	IVa	Lateral rhinotomy + R + C	Dead(3)
41	MS	Epistaxis(36)	III	ESS + R + C	Dead(18)
42	LNW,MS	Epistaxis(15)	IVa	ESS + R + C	Dead(32)
43	OC	Epistaxis(4)	III	Lateral rhinotomy + R + C	Dead(6)
44	NC	Nasal obstruction(3)	IVa	Lateral rhinotomy + R	Dead(24)
45	NC	Nasal obstruction(1)	III	ESS + R + C	Alive(60)
46	NS	Epistaxis(0.5)	III	ESS	Dead(15)
47	NC	Nasal obstruction(1)	IVa	Lateral rhinotomy	Dead(14)
48	NP	Nasal obstruction(2)	III	ESS + R	Alive(58)
49	MS,SB	Nasal obstruction(2)	IVb	ESS	Dead(30)
50	OC	Epistaxis(0.5)	III	ESS	Dead(24)
51	LNW	Epistaxis(4)	III	ESS	Dead(6)
52	NC,ES	Nasal obstruction(4)	IVa	ESS + R + C	Alive(44)
53	NC,MS	Epistaxis(1)	III	ESS + R	Dead(32)
54	NS	Dryness in nasal cavity(12)	III	ESS	Alive(40)
55	NC,ES,FS,SS	Epistaxis(5)	IVa	ESS + R + C	Dead(27)
56	NC	Epistaxis(2)	IVa	Midfacial degloving + R + C	Alive(38)
57	NC	Epistaxis(6)	IVa	Lateral rhinotomy	Dead(23)
58	NC	Epistaxis(6)	III	ESS + R + C	Dead(13)
59	NC	Epistaxis(2)	III	ESS	Dead(13)
60	LNW	Epiphora(60)	IVa	ESS + R	Alive(30)
61	LNW,ES	Epistaxis(6)	IVa	ESS	Dead(11)
62	NC	Epistaxis(2)	IVa	ESS	Dead(10)
63	NC	Epistaxis(3)	III	ESS + R + C	Alive(27)
64	NC	Nasal obstruction(2)	III	ESS	Dead(14)
65	NC,ES	Epistaxis(1)	IVa	ESS	Dead(8)
66	NC	Nasal obstruction(1)	IVa	ESS + R + C	Dead(27)
67	ES,MS,SS	Nasal obstruction(2)	IVa	ESS	Dead(22)
68	NC,MS	Epistaxis(2)	III	ESS + R + C	Dead(18)
69	LNW,MS	Nasal obstruction(1)	III	ESS	Dead(21)

*R* radiotherapy, *C* chemotherapy, *ESS* endoscopic sinus surgery, *m* male, *f* female, *LNW* lateral nasal wall, *NP* nasopharynx, *OS* olfactory cleft, *MS* maxillary sinus, *ES*:ethmoid sinus, *FS* frontal sinus, *SS* sphenoid sinus, *NC* nasal cavity, *PF* pterygopalatine fossa, *NS* nasal septum, *O* orbit, *SB* skull base.

### Treatment and survival outcomes

**Figure 2:**
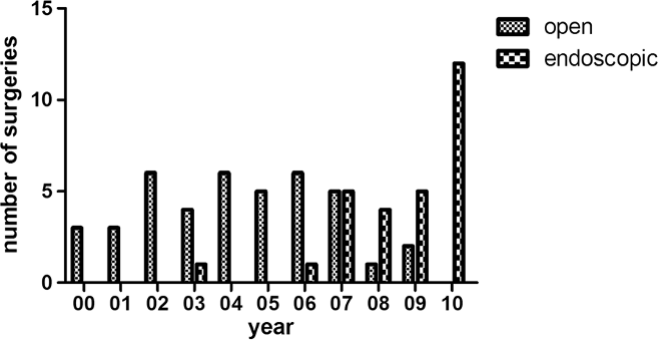
**Surgical approach distribution per year.**

Surgical approaches involved lateral rhinotomy, Caldwell-Luc, transpalatal, midfacial degloving procedure, and endoscopic approach. Forty one and 28 cases underwent open and endoscopic approaches, respectively. One patient underwent neck dissection because regional lymph nodal metastasis was identified. Surgical approach distribution per year was listed in Figure 2. Thirty percent of cases needed multiple surgeries during their course of disease, with a mean of 1.6 times (ranging from 1–7 times). Twenty seven patients required surgical treatments alone, 24 patients accepted postoperative radiotherapy, and 18 patients accepted triple treatment of surgery, radiotherapy plus chemotherapy. Commonly, patients were not positioned for adjuvant therapy because they either refused, were intolerant to adjuvant therapy or were considered to have a complete tumor resection based on the surgeon's assessment. Clinical data was summarized in Tables 2 and 3 for different surgical and adjuvant treatment modalities and approaches. Statistical difference was not found in age, gender or staging among different surgical treatment modalities and approaches except the gender between groups of surgery alone and surgery plus radiotherapy, more male patients underwent postoperative radiation treatment than female patients.

**Figure 3:**
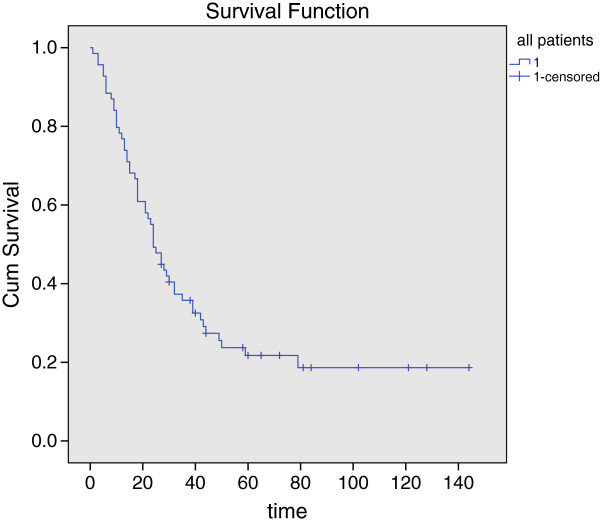
**Kaplan-Meier graph of overall survival of 69 SMM cases.**

The cut-off date for follow-up was December 31st, 2012. By that time, 53 patients had died and 16 patients had survived for their last follow-up. Seven patients had died of unassociated conditions, such as heart and liver diseases. The mean follow-up time was 34 months (ranging from 1–144 months). Generally, 42% patients (n = 29) were certain to develop local recurrence, 17% (n = 12) and 41% (n = 28) for nodal recurrence and distant metastasis, respectively. The median overall survival time of all patients was 24 months (standard error (SE) = 2.373, 95% confidence interval (CI) = 19.349-28.651). The estimated 3 and 5 year overall survival rates for all patients were 35.8% and 21.8%, respectively, as depicted in Figure 3. The median overall survival time was 24 months (SE = 4.204, CI = 15.760-32.240) for male patients; and 24 months (SE 6.364, CI = 11.527-36.473) for female patients. There was no statistical significance reached between genders (P = 0.706).

**Figure 4:**
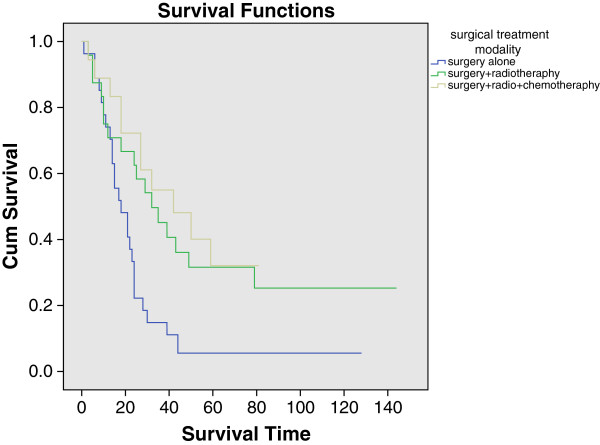
**Kaplan-Meier graph of overall survival comparison among different treatment modalities.**

The median overall survival time was 18 months (SE = 3.894, CI = 10.367-25.633) for surgery alone, 32 months (SE = 8.124, CI = 16.077-47.923) for surgery plus radiotherapy, and 42 months (SE = 14.749, CI = 13.092-70.908) for surgery, radiotherapy plus chemotherapy. The 3 and 5 year overall survival rates for the groups mentioned above were 14.8% and 5.6%, 45.1% and 31.6%, 55% and 32.1%, respectively. Significance was found not only between surgery alone vs. surgery plus radiotherapy treatment modality (P = 0.012), but also surgery alone vs. surgery, radiotherapy plus chemotherapy modality (P = 0.002). There was no statistical difference between groups of surgery plus radiotherapy and surgery, radiotherapy plus chemotherapy (P = 0.601). Associated survival was graphed in Figure 4.

**Table 2:** **Summary for clinical data of different surgical treatment modalities**

Treatment(group number)	Age of patients median(range)	Gender of patients			Stage of disease				Median survival months(SE)(CI)
		Male	Female	Total	III	IVa	IVb	Total	
Surgery alone(1)	69(28–89)	19(70%)	8(30%)	27	15(56%)	10(37%)	2(7%)	27	18(3.894)(10.367–25.633)
Surgery + radiotherapy(2)	63(38–88)	8(33%)	16(67%)	24	13(54%)	10(42%)	1(4%)	24	32(8.124)(16.077–47.923)
Surgery + radiotherapy + chemotherapy(3)	65(45–79)	10(56%)	8(44%)	18	9(50%)	7(39%)	2(11%)	18	42(14.749)(13.092–70.908)
P value(group1 vs. group2)	0.152	0.012			1.000				0.012
P value(group1 vs. group3)	0.310	0.354			1.000				0.002
P value(group2 vs. group3)	0.712	0.211			0.791				0.601

**Table 3:** **Summary for clinical data of different surgical approaches**

Surgical approach	Age of patients median(range)	Gender of patients			Stage of disease				Median survival months(SE)(CI)
		Male	Female	Total	III	IVa	IVb	Total	
Open approach	66(38–89)	19(46%)	22(54%)	41	20(49%)	17(41%)	4(10%)	41	24(2.554)(18.993–29.007)
Endoscopic approach	65(28–89)	18(64%)	10(36%)	28	17(61%)	10(36%)	1(3%)	28	27(5.170)(16.866–37.134)
P value	0.753	0.219			0.517				0.687

The median cause-specific survival time was 21 months (SE = 3.450, CI = 14.238-27.762) for surgery alone, 35 months (SE = 10.055, CI = 15.291-54.709) for surgery plus radiotherapy, and 42 months (SE = 14.749, CI = 13.092-70.908) for surgery, radiotherapy plus chemotherapy. The same statistical significance was found as the overall survival comparison among these groups: (sa vs. sr, P = 0.041; sa vs. src P = 0.011; sr vs. src, P = 0.593).

**Figure 5:**
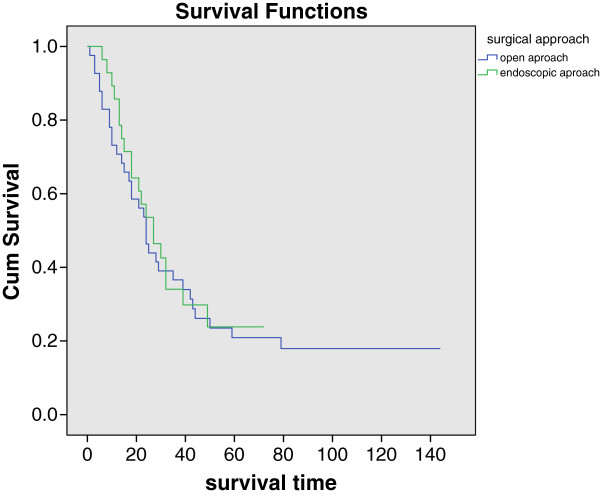
**Kaplan-Meier graph of overall survival comparison between open and endoscopic approaches.**

The estimated 3 year local control rate was 25.3% for surgery alone, 48.7% for surgery plus radiotherapy, and 42.9% for surgery, radiotherapy plus chemotherapy. The estimated 3 year nodal control rate was 51% for surgery alone, 71.6% surgery plus radiotherapy, and 56.3% for surgery, radiotherapy plus chemotherapy. The estimated 3 year distant metastasis-free rate was 38.6%, 53.6% and 55% for groups mentioned above respectively. The median disease-free survival time was 11 months (SE = 2.077, CI = 6.929-15.071) for surgery alone, 16 months (SE = 4.882, CI = 6.431-25.569) for surgery plus radiotherapy, and 16 months (SE = 2.121, CI = 11.842-20.158) for surgery, radiotherapy plus chemotherapy. There was no statistical significance reached for above survival comparisons among these groups. In regards to different surgical approaches, median overall survival time was 24 months (SE = 2.554, CI = 18.993-29.007) for open approach and 27 months (SE = 5.170, CI = 16.866-37.134) for endoscopic approach. The 3 and 5 year overall survival rate were 36.6% and 20.9% vs. 34% and 23.8% for each group mentioned above respectively. There was no survival statistical difference between the two groups (P = 0.687). Associated survival was graphed in Figure 5.

The median cause-specific survival time was 25 months (SE = 8.147, CI = 9.031-40.969) for open approach and 27 months (SE = 4.712, CI = 17.764-36.236) for endoscopic approach. The median disease-free survival time was 14 months (SE = 2.680, CI = 8.748-19.252) for open approach and 15 months (SE = 2.639, CI = 9.828-20.172) for endoscopic approach. There was no statistical significance reached for cause-specific survival (P = 0.989) and disease-free survival (P = 0.899) comparion between these two groups.

## Discussion

Sinonasal malignant melanoma is a rare malignant tumor with very a poor prognosis. Most patients died within two years after diagnosis. For SMM, a randomized controlled trial is difficult to perform because of its poor prognosis and rare incidence. Therefore, most studies published were case reports or retrospective analysis of series of patients over many decades which may have been affected by the development of other co-morbid or unrelated medical conditions. Accordingly, it is difficult to be certain which therapeutic strategy is the most optimal. We analyzed data of patients over a span of 11 years which may have avoided some of the potential effect of other medical conditions. Also owning to its rarity of SMM, it would be difficult to perform a comparative study with a large sample size (in a relatively shorter time span) and most published studies were based on malignant tumors with heterogeneous histopathologic diagnosis [11, 12]. Since tumors with different histopathologic characteristics may have their own biological behavior which may differ greatly to a homogeneous SMM, it would be more revealing to study a group of histopathologically more uniform tumors. To our knowledge, our study is the largest series from a single center with homogeneous histopathologic malignant melanoma in about one decade period of time.

### Prognosis & therapeutic selection of SMM

From our study, we found that the 3 and 5 year overall survival rates of all patients were 35.8% and 21.8% respectively. This is similar to most other studies [6, 13–18]. Poor prognosis may be due to the frequent recurrence and distant metastasis at an early stage, despite radical surgeries performed. It was reported that the postoperative time for local recurrence presentation was 19.8 months and even 12.3 months for distant metastasis [19]. The local recurrence and distant metastasis rate was 31-85% and 25-50%, respectively [20–23]. In our cohort, 42% patients were certain to develop local recurrence, 17% and 41% for nodal recurrence and distant metastasis, respectively. Though surgical resection is considered the mainstay therapy, tumor resections with clear negative surgical margins were frequently impossible due to the anatomical complexity and the adjacent vital structures of the sinonasal region. Thus, adjuvant therapy was frequently given following surgery. In localized patients, surgery alone or surgery in combination with radiotherapy has been advocated as definitive therapy. Despite the fact that malignant melanoma is considered as radioresistant, radiation therapy would be planed if complete surgical excision is not possible due to the extent of disease at presentation [2, 24].

The role of adjuvant therapy like radiotherapy and chemotherapy is still controversial. Gore did a meta-analysis on the survival of sinonasal melanoma, and demonstrated that multimodality therapy, particularly the addition of chemotherapy or immunotherapy to surgery, might increase survival in a subset of patients; while radiation therapy did not appear to increase survival. There may be a significant increase in the overall survival rate with combined modality therapy including surgery and chemotherapy or immunotherapy versus single modality therapy [9]. Kanetaka found immunotherapy using LAK cell treatment may contribute to the improvement of the prognosis in patients with malignant melanoma in the head and neck [25].

Kingdom and Kaplan reported patients who received postoperative radiotherapy appeared to have an increase in disease-free intervals and prolonged survival [26]. Evidences have also shown that radiotherapy could improve local control [7, 8, 18], but had no effect on overall survival, while others found little effect from postoperative radiotherapy [6, 9, 11, 20, 27]. Given the rarity and poor prognosis of SMM, radiotherapy groups in most studies involved patients who had extensive and unresectable tumors and were at more advanced stages. The unfavorable results of combined surgery and radiotherapy regimen might be related to much earlier distant metastasis and thus more advanced staged patients in postoperative radiotherapy groups.

We intended to analyze the impact of different surgical and postoperative adjuvant therapeutic modalities on survival, therefore all patients in our study were selected from surgical treatment pool and inoperable cases had been excluded. In our series, we did not find any statistical difference in age or stage among different surgical treatment modalities selected. Those patients who underwent radiotherapy or radiotherapy plus chemotherapy after surgery had better overall and cause-specific survival outcomes than those who accepted surgical treatment alone. There was no statistical difference between the former two modalities. In our cohort, patients who underwent radiotherapy or radiotherapy plus chemotherapy after surgery were also found to have a better local (nodal) control rate as well as a distant metastasis free rate, than those accepted surgical treatment alone. Although comparison regarding local (nodal) control status as well as distant metastasis free status among groups above did not revealed any statistical significance, adjuvant therapy is necessary to achieve maximal tumor eradication, because often the complete tumor resection with clear margin is unobtainable. Reports have shown that despite adequate local control, there were patients who still suffered frequent distant metastasis. Sinonasal melanoma is difficult to control as distant spread is likely to occur even in the presence of mainstay locoregional control [9]. Thus, future, more rigous, multi-center studies should be performed to further investigate the effect of combined therapeutic strategies against this aggressive malignancy.

### Endoscopic approach for treatment of SMM

The optimal therapeutic results can be achieved with radical tumor resection with wide negative margins. Complete resection requires the inclusion of at least 1.5 cm of normal tissue margins, as well as cervical lymph node dissections [3].

With the assistance of nasal endoscopy, better surgical optical visualizations were obtained than an open approach and in many cases the open surgical approach can be replaced by the endoscopic one. The endoscopic approach allows for intraoperative navigation to determine the border and resection margins of tumor. Doppler ultrasound is also a useful technology to ascertain the location of the internal carotid artery in order to avoid disastrous complications. Suh et al. reported fewer major surgical and medical complications in endoscopic resection of sinonasal malignancies vs. open approaches [11]. Moreno et al. found a higher rate of survival for patients who underwent endoscopic tumor surgeries, which might reflect a selection bias as this approach was more commonly used for lesser volume and more localized disease [19]. Vandenhende et al. found no difference in local control of T4 maligant lesions, comparing external versus endoscopic approaches [20]. We also compared open and endoscopic approach for treatment of SMM, and endoscopic approach had similar survival status as open approach which is in accordance with most other studies, without any statistical difference between two groups. Endoscopic tumor resection has many advantages such as minimally invasiveness, direct tumor exposure, more optimal post-operative cosmetic appearance, fewer complications and shorter hospital stays [11, 12]. When prudently selected, endoscopic approach might be the first choice for SMM surgery.

### Weakness and deficiencies of our study

Like many others in the literature on SMM, our study is also a retrospective study which would make the results less valuable comparing to those randomized prospective controlled trials. Also due to the retrospective nature, we were not able to acquire the quality of life data (before and after surgery) from patients, which may be meaningful to reflect the potential advantage of endoscopic approach over open ones. Furthermore, we were not able to retrieve complete pathological information about surgical margins. Thus, it was difficult to analyze the impact of surgical margins on prognosis. Data from a single center may contain selection bias on its acceptance criteria for surgical and adjuvant treatment. Further multi-center or even population-based series would be the better model for studies of rare malignant tumors such as sinonasal malignant melanoma.

## Conclusions

Sinonasal malignant melanoma is a disease with a poor prognosis. Patients who underwent surgery plus radiotherapy or surgery, radiotherapy plus chemotherapy had better survival outcomes than those who underwent surgery alone. The endoscopic surgical approach had similar survival outcomes as compare to the open surgical approaches.

## Author’s information

Xin-Jun Meng Attending doctor at Ruijin Hospital, School of medicine, Shanghai Jiao Tong University; Now as a PhD candidate at Eye Ear Nose and Throat Hospital, Fudan University

## Abbreviations

SMM: Sinonasal malignant melanoma
SE: Standard error
CI: Confidence interval
R: Radiotherapy
C: Chemotherapy
ESS: Endoscopic sinus surgery
m: Male
f: Female
LNW: Lateral nasal wall
NP: Nasopharynx
OS: Olfactory cleft
MS: Maxillary sinus
ES: Ethmoid sinus
FS: Frontal sinus
SS: Sphenoid sinus
NC: Nasal cavity
PF: Pterygopalatine fossa
NS: Nasal septum
O: Orbit
SB: Skull base.
